# Status and Prospect of Ecological Environment in the Belt and Road Initiative Regions

**DOI:** 10.3390/ijerph192417091

**Published:** 2022-12-19

**Authors:** Xixi Du, Yi Qin, Chunbo Huang

**Affiliations:** 1 School of Foreign Languages, China University of Geosciences, Wuhan 430074, China; 2 Center for Turkmenistan Studies, China University of Geosciences, Wuhan 430074, China; 3 State Key Laboratory of Biogeology and Environmental Geology, School of Geography and Information Engineering, China University of Geosciences, Wuhan 430074, China

**Keywords:** the Belt and Road Initiative (BRI), ecological environment, eco-environmental protection, green development

## Abstract

With the widespread recognition and in-depth implementation of the Belt and Road Initiative (BRI), especially in the context of global climate change, the ecological environment of Belt and Road Initiative regions might be confronted with pressures and challenges with rapid socioeconomic development. In response to those potential environmental challenges, China has put forward Green BRI and enriched the new Silk Road with more environmental connotations, aiming to reduce the conflict between economic development and eco-environmental protection. Currently, there is a lack of systematic and holistic research on eco-environmental issues in BRI regions. In addition, feasible solutions to enhance BRI’s contribution to the eco-environment remain insufficient. Having systematically reviewed the relevant literature on the eco-environment in BRI regions, we found that most regions along the BRI routes are in sensitive zones of climate and geological change, with fragile eco-environments and strong vulnerability to climate change, natural disasters and human activities. The main eco-environment status of the BRI regions is as follows: (1) The total water resources in BRI regions account for only 36% of the global total, with uneven distribution and complex spatial precipitation, posing higher pressure on water security. (2) Vegetation varies significantly from region to region. The vegetation in South Asia is the richest, with its mean annual NDVI exceeding 0.7. The NDVI in East Europe, Russia and South China are between 0.4 and 0.7, and that in Central Asia and West Asia are below 0.2. (3) The BRI regions are abundantly blessed with natural resources, with the total recoverable oil reserves, natural gas reserves and the total mining area reaching 66%, 65.5% and 42.31% of the world’s total, respectively, but severe overexploitation and overconsumption of those resources degrade their eco-environment. Accordingly, future research directions, such as target on integrated, interdisciplinary and coordinated studies on eco-environmental issues in BRI regions, are proposed in this paper to achieve optimization of BRI’s contribution to eco-environment protection in BRI regions.

## 1. The Development of Belt and Road Initiative

The ancient Silk Road, established in about 140 B.C. during the Han Dynasty to promote trade between China and the West [1], provided a stimulus for cultural growth and economic prosperity along its entire route [2], making itself an “economic bridge” and a “cultural bridge” between the East and West. Against the backdrop of rapid global economic development in the new era, strengthening regional cooperation is an important driving force for world economic development. Therefore, to carry forward the ancient Silk Road’s centuries-old spirit of peace, friendship, inclusiveness, openness, mutual benefit and mutual learning, the ambitious long-term regional cooperation framework “Belt and Road Initiative (BRI)” was proposed, with a novel significance of cooperation [3].

On September 7, 2013, Chinese President Xi Jinping successfully proposed the construction of the “Silk Road Economic Belt” during his visit to Kazakhstan, Central Asia [4]. The same year, on October 3, President Xi delivered a speech entitled “Jointly Building a China-ASEAN Community of Shared Future” in Indonesia, Southeast Asia, proposing to build the “21st Century Maritime Silk Road” [5] jointly. These proposals, the “Silk Road Economic Belt” and “21st Century Maritime Silk Road”, are officially termed the “Belt and Road Initiative” [6], the specific routes of which are exhibited in Table 1 [7].

Since first proposed in 2013, the BRI has attracted an enthusiastic response from an increasing number of countries. As China moved to articulate the new policy with the publication of a 2015 White Paper, Oceania was naturally integrated into the existing network of trade corridors with the addition of the “Southern Leg Maritime Silk Road” [8]. During the Belt and Road Forum for International Cooperation in May 2017, President Xi Jinping responded for the first time during a meeting with President Mauricio Macri of the Republic of Argentina to include Latin America as a “natural extension” of the “21st Century Maritime Silk Road” [9,10]. Covering Asia, Europe, Africa, Oceania, South America, North America and the Caribbean, the BRI could be defined as the world’s largest platform for cooperation on public goods. The populations and GDPs of participating countries account for 63% and 31.79% of the global total, respectively [11]. It is estimated that, by 2060, the population will witness an increase to 1.83 billion, and GDP will increase by 3.0–6.4 times in BRI participating countries compared to 2016 [12]. Jointly building the Belt and Road is becoming an ambitious plan to promote global openness and cooperation, improve the global economic governance system, stimulate common development and prosperity of the world, and accelerate the building of a community with a shared future for mankind [13,14].

With the growth of BRI infrastructure construction projects and more frequent trade flows among BRI-involved countries, economic development may lead to overall environmental degradation [15]. To bolster the environmental credentials and reduce the negative environmental impact, in 2015, the Chinese government proposed the “Green Silk Road”, commonly known as the Green BRI, to facilitate BRI-involved countries to pursue environmentally conscientious behavior and implement the integration of eco-environment and development of the SDGs [16], making the BRI carry more significance. Under the theme of peace and development, the BRI aims to develop economic cooperative partnerships with countries along the routes, with policy coordination, infrastructure connectivity, unimpeded trade, financial integration and person-to-person bonds as its main goals, building a community of shared interests, responsibilities and destiny featuring political mutual trust, economic integration and cultural inclusiveness [6,17]. Through joint efforts, the BRI will be built into an initiative of peace, prosperity, openness, green development, innovation and civilization. In 2021, the non-financial direct investment of Chinese companies in BRI participating countries reached RMB 130.97 billion, growing by 6.7% year-on-year (USD 20.3 billion, up 14.1%). This sum accounted for 17.9% of their total outbound investment over the same period, up 1.7 percentage points from the previous year [18].

## 2. Temporal Variation of BRI-Involved Countries

Since first proposed in 2013, the BRI has attracted acute attention from countries around the world. By 23 March 2022, China had signed more than 200 cooperation documents on Belt and Road Cooperation with 149 countries and 32 international organizations, with 38 countries from Asia, 52 countries from Africa, 27 countries from Europe, 11 countries from Oceania, 9 countries from South America and 12 countries from North America and the Caribbean [19] (Table 2).

**Figure 1:**
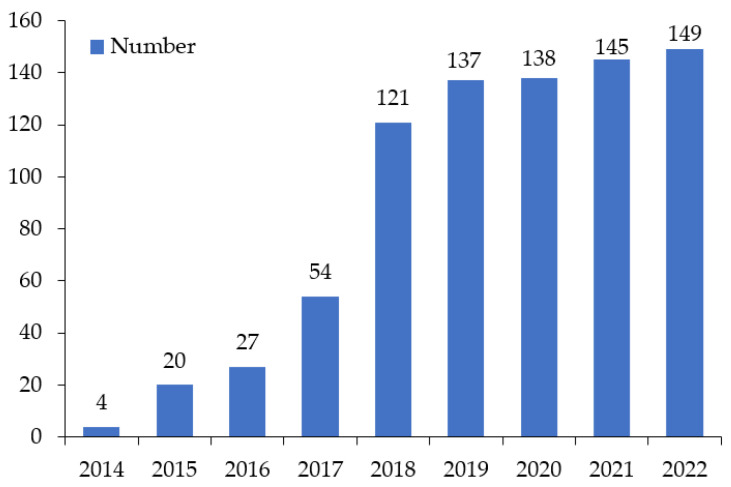
Dynamics of the annual number of BRI-involved countries from 2014 to 2022. (Source: the authors’ calculation based on the data from Belt and Road Portal).

The number of BRI-involved countries increased significantly from 2014 to 2022 (Figure 1). In 2014, in the early stage of the implementation of the BRI, there were only four countries signed a Memorandum of Understanding (MoU) for cooperation on the BRI, namely Kazakhstan, Belarus, Sri Lanka and Qatar. However, with the aim to create the world’s largest platform for economic cooperation, including policy coordination, trade and financing collaboration, and socioeconomic cooperation [17], the BRI promoted win-win cooperation for shared development, prosperity, peace and friendship [20], attracting an increasing number of countries and organizations to involve. The year 2018 witnessed the largest increase in the number of countries signing the MoU, from 54 to 121.

**Figure 2:**
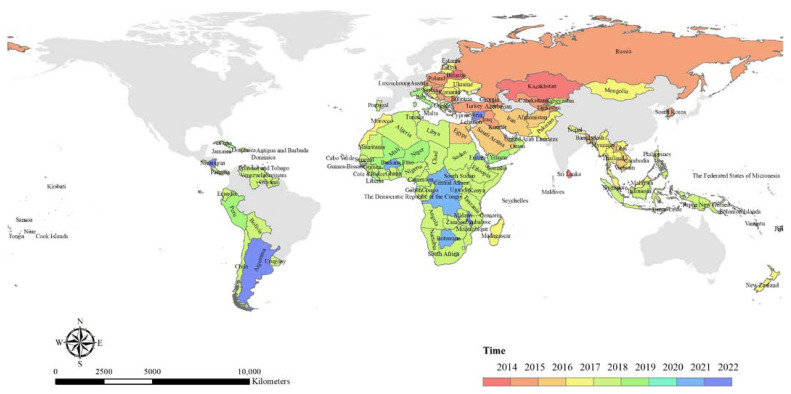
A visualized map of spatiotemporal variation of BRI-involved countries from 2014 to 2022. (Source: created by authors by ArcGIS according to Table 2).

According to the visualized map of the spatiotemporal variation of the BRI-involved countries (Figure 2), the countries signing the MoU were mainly from Asia and Europe in the early stage of the implementation of the BRI. In 2018, a large-scale expansion appeared in Africa. Subsequently, an enormous number of Oceania and Latin American countries have been involved in the BRI since 2018.

## 3. Classification of the BRI-Involved Countries

Consisting of two components—a terrestrial “belt”, referring to the “Silk Road Economic Belt”, connecting China with Central Asia, Western Asia, Russia and Europe, and a maritime “road”, referring to the “21st Century Maritime Silk Road”, linking Chinese coastal ports with those in Southeast Asia, South Asia, Africa, the Middle East and Europe [7]—the BRI envisions a vast network of infrastructure projects including the construction of railways, highways, ports and pipelines, as well as the stimulation of industrialization, energy and resources development, etc. [21]. In 2015, Oceania was formally integrated as one of China’s routine trading partners with the establishment of the “Southern Leg Maritime Silk Road”, a natural extension branch of the wider BRI [22]. In March 2017, New Zealand signed an MoU for cooperation on the BRI, becoming the first Oceanian country to participate in the BRI [23]. Subsequently, a growing number of Oceanian countries were attracted to participate in the BRI. Additionally, Latin America has been officially included as a “natural extension” of the Maritime Silk Road and an “indispensable participant” of the BRI since the holding of the 2017 Belt and Road Forum [24]. The Silk Road Economic Belt and the 21st Century Maritime Silk Road overlap in Europe, however, with most European countries located in the Silk Road Economic Belt region, European countries are catalogued into the Silk Road Economic Belt region in this study. Therefore, the BRI-involved countries could be classified into three main parts, which are scattered in the Silk Road Economic Belt region (40), the 21st Century Maritime Silk Road region (77) and the natural extension region of the Maritime Silk Road (32), respectively. 

**Figure 3:**
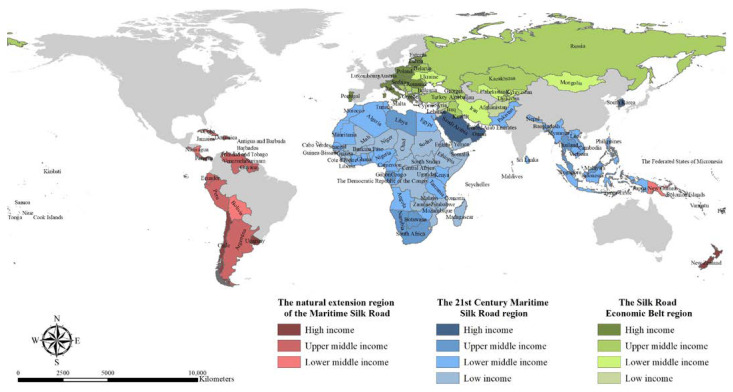
The distribution map of the BRI-involved countries of the three corresponding regions by different income groups in 2022. (Source: created by authors by ArcGIS according to Table 3).

An overview of the countries of the three BRI composing regions is exhibited with a detailed classification of their income levels (Table 3) [25]. The income group classification refers to the World Bank categorization (https://data.worldbank.org/country, assessed on 22 July 2022). Meanwhile, the distribution of the BRI-involved countries of the three corresponding regions by different income groups is visualized (Figure 3). The countries belonging to high-income groups are mainly concentrated in Europe. The 21st Century Maritime Silk Road region contains the largest number of lower-middle-income and low-income countries, mainly in central Africa. As for promoting sustainable economic growth, BRI-participating countries with low income and lower-middle income benefit more than countries with high income and higher-middle income [25]. Participating in the BRI is an excellent opportunity for developing countries with relatively low incomes to stimulate economic growth through a green and environment-friendly development path [26]. In addition, there are no low-income group countries in the natural extension region of the Maritime Silk Road.

## 4. Ecological Environment Status in Belt and Road Initiative Regions

### 4.1. Overall Ecological Environmental Patterns

The Belt and Road region traverses Eurasia, Africa, Oceania, South America, North America and the Caribbean and spans subtropical, temperate, cold temperate and frigid zones, with complex terrain and geological conditions [27,28]. There are many ecologically sensitive areas, including biodiversity hotspots and protected areas [29]. Among them, Southeast Asia, the most biodiverse region in the world, boasts a large number of protected areas and biodiversity hotspots [30]. With ASEAN countries partnering with China to enhance regional economic growth and promote close trade with China, Southeast Asia is increasingly becoming a hotspot for infrastructure development under BRI. BRI-related infrastructure traverses through Southeast Asia via various transportation corridors, which could pose detrimental challenges to the eco-environment of the region. Since the implementation of the BRI, a total of 21 terrestrial protected areas, accounting for 4% of 472 protected areas, in mainland Southeast Asia have been directly bisected by the BRI routes which traversed through 210 km of terrestrial protected habitat. Meanwhile, a total of 20 marine protected areas and 16 marine key biodiversity hotspots in insular Southeast Asia have been potentially affected within 50 km of the BRI’s marine route [31].

Over 60% of the Belt and Road region areas are arid and semi-arid grassland, desert and high-altitude ecologically fragile areas with a dry climate and low precipitation [27], having a strong vulnerability and relatively low adaptive capacity to climate change, natural disasters and human activities [32,33]. The total water resources in Belt and Road region are only 36% of the global total, with an uneven distribution, posing higher pressure on water security compared with the world average level [27]. The highest water resources per capita among the Belt and Road region were found in South and Central America, with an average of 39,901 m^3^·yr^−1^ compared with the lowest of 340 m^3^·yr^−1^ in North Africa [34]. The spatial precipitation of the main water resource area of the Belt and Road region had complex conditions (Table 4) [35].

Due to rare precipitation and intensive evaporation, the region from northwest China to Western Asia and North Africa is confronted with severe water shortages [35]. Additionally, there are seasonal variations in drought among the Belt and Road region, with the area of winter drought larger than that of summer drought [36]. Owing to the fragile natural environment and frequency of disasters, the Belt and Road regions are vulnerable to climate extremes and natural disasters, including floods, droughts, landslides, wildfires, tsunamis, typhoons, heat waves, convective storms, cold temperatures and earthquakes, which inflict severe damage on society, economy, ecosystems and local residents’ daily lives at global and regional scales [32,37,38,39,40,41,42,43], and the frequency, duration and intensity of these are likely to increase continuously [44]. The regional characteristics of natural disasters in the Silk Road Economic Belt region are exhibited in Table 5 [35,39,41,45,46].

The area of the Maritime Silk Road, subject to the tropical monsoon climate, is one of the areas with the most frequent marine and meteorological disasters, such as typhoons, storm surges, severe waves and tsunamis [42]. The regions affected by storm surges are mainly distributed in East Asia, Western Europe and northern Australia. In addition, the sources of major tsunamis are the several large tectonic faults on the Maritime Silk Road, most of which are located in the northwestern Pacific Ocean and the northeastern Indian Ocean, making tsunami events mainly distributed in the South China Sea, the eastern Indian Ocean, the Arabian Sea and the Mediterranean coast [37,42]. Meanwhile, human activities, such as rapid urbanization, often result in abrupt land-use changes that lead to eco-environment degradation, including vegetation reductions, increased coastal erosion and reduced ecosystem diversity [47]. Given that the Belt and Road regions are mainly ecologically fragile areas with complicated, diverse and vulnerable eco-environmental conditions [48], the eco-environmental issues along the BRI regions are worthy of in-depth study for the future formulation of countermeasures and eco-environmental protection strategies. However, the existing literature predominantly centers on Eurasia and Africa, and it has not been extended to the expanded areas of the BRI, such as Oceania, South America, North America and the Caribbean. In the future, the scope of research should be further extended to enrich systematic and integrated research on the eco-environment of the BRI regions.

### 4.2. Vegetation Coverage Conditions

Terrestrial vegetation enacts an important role in ecosystems, such as mitigating global warming, preventing soil erosion and alleviating city heat islands [47,49,50]. Coastal vegetation, including mangroves, salt marshes, macroalgae, seagrasses, coastal strands and dunes, also buffer shores and retain sediments from tides, waves and storms [47]. However, the vegetation coverage in the Belt and Road region has experienced a significant change in recent years. The normalized difference vegetation index (NDVI) of the whole region revealed a slow decrease during 1982–2015, with decreased vegetation NDVI mainly distributed in northern Russia, Central Asia (the coast of the Black Sea, Caspian Sea and Aral Sea), Southeast Asia, the Malay Islands and northeast China, of which, the magnitude of the decrease in the north of Russia is particularly remarkable. Additionally, several areas witnessed an increase in vegetation NDVI, mainly concentrating in East Asia, Europe and China [33]. The mean annual NDVI of some regions is shown in Table 6 [51].

From 1981 to 2016, especially after 2005, vegetation trend shifts existed in Belt and Road region. The greening to browning shifts were the most common category, accounting for 23.23% of the vegetated area, primarily in eastern Europe and Central Asia, with warmer temperatures, droughts, land abandonments and agriculture expansions as the main causes [51,52,53]. Additionally, economic development and trade activities brought by the BRI are some of the main driving forces behind land use and land cover changes (LUCC), which affect the vegetation coverage. For instance, the impact of export trade under BRI on LUCC in Central Asia before and after the implementation of BRI were evaluated, the results of which showed that agricultural land and construction land increased by 59,120 km^2^ and 7617 km^2^, respectively, while ecological land decreased by 66,737 km^2^ before and after the BRI (2001–2020). Overall, the development of trade under BRI may affect the changes in ecological land and reduced vegetation coverage in Central Asia [54].

Most of the countries in the Belt and Road region are developing countries in semiarid and arid areas with relatively lower income levels, high rural poverty and heavy environmental threats, making its vegetation highly sensitive to climate change and anthropogenic activities over multiple timescales [52,55,56,57]. Climate change dominates the overall vegetation coverage change in Belt and Road region, with warmer temperatures and droughts the likely main causes of the greening to browning shifts [52,58]. Moreover, anthropogenic activities such as deforestation, agricultural cultivation, and unplanned urbanization have dramatically changed vegetation at short time scales [57]. There is a substantial body of scholarly research focusing on vegetation change and its driving factors in the field of vegetation coverage in BRI regions; however, the literature on ecological restoration in vegetation loss areas is surprisingly scant. Accordingly, how to propose countermeasures to the factors causing vegetation degradation under the framework of the BRI and restore vegetation deserves to be intensively studied.

### 4.3. Climate Change Effects

Due to the complex climatic conditions, countries along the Belt and Road have experienced increasingly severe climatic extremes in recent years and are sensitive and vulnerable to climate change [28,59,60,61,62]. With its fragile ecosystem, poor infrastructure, relatively low income and high vulnerability to climate change, the Belt and Road region has experienced accelerated warming at roughly twice the rate of the global land [61,63]. In the context of climate change, rising temperatures have further exacerbated water stress in the Belt and Road region by increasing surface evapotranspiration and stimulating considerable glacial retreat, especially in Central and West Asian countries, which are currently facing water shortage problems [34,55,60,61,64]. Furthermore, climate change might make future water resources more unevenly distributed among the Belt and Road region (Table 7) [34].

With increased temperature, intensified water stress and changes in precipitation patterns, drought in the Belt and Road region has become more severe, leading to global desertification [61]. Potential changes in the usage of limited water resources may further increase the risk of desertification and deepen the area’s ecological poverty [64]. From 1986 to 2005, the annual aridity index in the Belt and Road region was approximately 1.58 [59]. The increased temperature might result in persistent drought intensification. With the effects of global warming, under the 1.5 °C global warming scenario, the aridity index in Central-Eastern Europe, north of West Asia, south of East Asia and northwest of Southeast Asia will rise rapidly at a rate of over 5% compared to 1986–2005, and under a global warming scenario of 2.0 °C, the aridity index in the same regions will further grow by 15% [59]. Additionally, the intensification of drought stress caused by rising temperatures and decreasing precipitation seriously leads to prominent vegetation browning in the Belt and Road region, especially in Central Asia [61].

Countries along the 21st Century Maritime Silk Road and the natural extension region of the Maritime Silk Road will witness a significant sea level rise [28]. Central Asia and South Europe are suffering from record extreme weather and climate events, which have resulted in huge economic losses and casualties. Southeast Asia, North Asia and South Asia are experiencing heavy precipitation events and extreme heat waves [28,40]. As for the future effects of climate change, it is predicted that Nairobi, the capital of Kenya (in East Africa), will experience the greatest increase in annual mean precipitation. Meanwhile, precipitation-related extreme events, such as drought and flooding events, are predicted to be intensified in Belt regions due to future extreme climate changes, particularly over West Asia and Southeast Asia [32].

As for the driving forces behind climate warming, CO_2_ emissions play an important role in accelerating climate warming [65]. Therefore, there are many studies using CO_2_ emissions as a proxy variable to estimate the impact of the BRI on the eco-environment under climate warming scenarios. Nevertheless, the results produced in those studies were inconsistent and even contradictory. For instance, regarding the effect of trade openness and foreign direct investment (FDI) on CO_2_ emissions, some researchers indicated that trade openness and FDI between China and the BRI countries significantly reduce CO_2_ emissions [66,67,68], whereas other researchers demonstrated that trade openness and FDI are significant contributors to CO_2_ emission in the BRI countries [26,69]. Still, some researchers showed that the BRI countries’ imports from China significantly reduce CO_2_ emissions in these countries and exports to China tend to promote CO_2_ emissions [67,70]. Other scholars classified the BRI countries according to their income levels, exploring the impact of imports and exports on CO_2_ emissions in different categorized BRI countries [26]. Results have shown that imports increased CO_2_ emissions in low-income countries while decreasing them in middle- and high-income countries, and exports decreased CO_2_ emissions in low- and high-income countries while increasing them in lower-middle countries. In a word, to evaluate the impact of the BRI on the eco-environment accurately, studies on the mechanisms for grouping BRI countries need to be carried out, so as to conduct more targeted research on the CO_2_ emissions solutions for each country along the BRI routes. Meanwhile, previous research only presents what happened in the past and predicts that those trends will continue in the long run. Future research may adopt a more dynamic view via scenario development and modeling.

### 4.4. Natural Resources Conditions

Natural resources are the foundation of human development, and the supply-consumption relationship of natural resources reflects the most basic impact of human activities on the ecosystem [12,71]. The BRI participating countries, with petroleum exporting and emerging economies in the list, are the major suppliers of natural resources and manufacturers of commodities [63], which boast abundant natural resources, including water resources, mineral resources, fossil energy (such as coal, crude oil and natural gas), and forest resources [72,73,74]. The statistics in Table 8 demonstrate the types and contents of mineral resources in the Belt and Road region [69,75,76]. Moreover, the copper reserves of the BRI-related countries comprise 396 million tons, accounting for about 47.71% of the world’s copper reserves, mainly distributed in Myanmar, Russia and Indonesia [77].

On the whole, the environmental opportunities for resource endowment in northern and southern regions are higher than in the middle region in the Belt and Road region [73]. Middle Eastern, North African and Central Asian countries are rich in mineral resources and energy, such as oil and natural gas, but are short of water and forest resources [73,75]. The Middle East and West Asia are the world’s largest oil reserve regions with the highest amount of oil produced and exported [74]. However, water resources there are extremely scarce, which presents a striking contrast to the richness of the oil. Central Asia is rich in mineral resources and fossil energy, especially Kazakhstan. Nevertheless, the northwestern part of Central Asia struggles with infrequent rains, poor soils and a lack of vegetation cover [73]. With the similar natural endowment condition, Africa is also rich in mineral resources but short of water resources, facing problems in the areas of water environmental protection and water supply security [47,78,79]. By contrast, Southeast Asia, with abundant water, forest, and mineral resources, has the most notable natural resource endowment [73]. Different countries boast their own abundant types of resources with different resource endowments. Nonetheless, few scholars have addressed the issue of resource complementarity and supply demand relationship among BRI regions. Therefore, making full use of the BRI’s platform advantages to avoid overexploitation of resources in a certain region and achieve resource complementarity via multilateral cooperation for different BRI-involved regions will provide a new angle for BRI resource research.

The total water resource distribution of the Belt and Road region is rich in the east and poor in the west, showing regional differences, with an imbalance of water resources exacerbating in this region [80,81]. Russia has the most abundant water resources, reaching 4525 billion m^3^, accounting for 23.09% of the total water resources of the Belt and Road region. The total water resources of China, India, Bangladesh, Myanmar, Indonesia and other countries all exceed 1 trillion m^3^, which is at a relatively abundant level. With relatively scarce water resources, the total water resources of countries in West Asia, Central Asia and the Middle East are generally less than 100 billion m^3^ [81]. Meanwhile, water for industrial and agricultural production accounts for the vast majority of water usage, water utilization efficiency of which has a significant impact on water resource utilization [78,82]. Uneven distribution of water resources, large utilization of water resources and low utilization efficiency will lead to serious water shortage and stress in the Belt and Road region [78,82,83]. Most countries in Belt and Road region are experiencing different degrees of water scarcity, with Central Asia being particularly prominent [83,84,85]. The BRI-related countries are all facing water crises but with different priorities. Therefore, when implementing cooperation projects under the framework of BRI, attention should be paid to the water shortage in West Asia, Central Asia and the Middle East, cooperation programs should be designed according to the water consumption situation in different regions, so as to improve the efficiency of local water resources utilization and ensure local water security.

**Figure 4:**
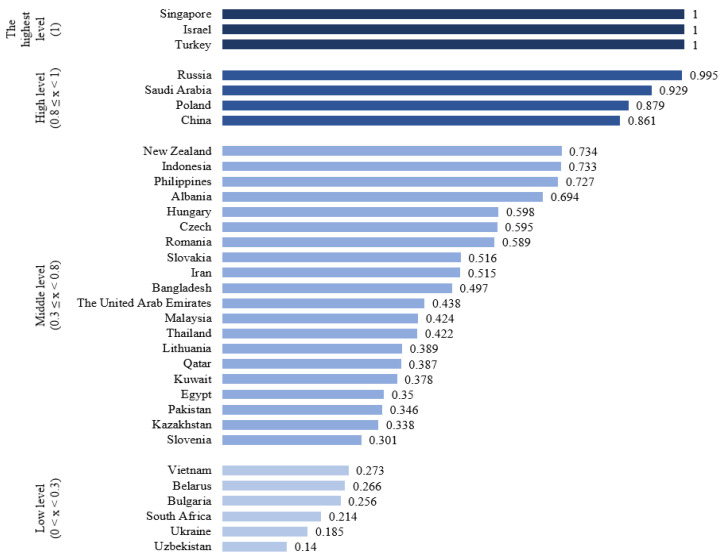
Total-factor energy efficiency (TFEE) in BRI participating countries. (Source: data from Ref. [87].).

Mineral resources are an essential material basis for socioeconomic development, and almost all industrial sectors have a connection with the consumption of mineral resources [86]. Rich in mineral resources, the BRI-related countries are a globally important supply base of resources, where mineral resource exploitation plays an irreplaceable role in their socioeconomic development [76]. In those countries with a high proportion of fossil energy, the problem of energy waste is severe and the total-factor energy efficiency (TFEE) varies from each other (Figure 4) [87].

In the future, improving energy management and technology utilization levels should be focused on reducing energy waste and improving resource utilization efficiency under the cooperation framework of BRI. Moreover, the overexploitation and overconsumption of mineral resources and the deposition of mining waste will lead to various secondary eco-environmental problems, such as vegetation degradation, land occupation, ground subsidence, and biodiversity loss, which will pose challenges to the regional eco-environment [86].

Natural resources are considered to be the essential elements of socioeconomic development. However, the overexploitation of natural resources in agricultural production, mining and deforestation may create eco-environmental issues [88]. Consequently, the usage of natural resources should be controlled, resource productivity and utilization efficiency should be improved and overexploitation and overconsumption should be avoided in the BRI regions.

### 4.5. The Role of BRI in the Context of the Ecological Environment

Since the implementation of the BRI, China’s cooperation with BRI participating countries has been growing rapidly. Due to the large-scale projects of BRI, BRI is a potential contributor to eco-environmental challenges for participating countries [89]. In the early stage of the implementation of the BRI, infrastructure development, trade and investments under the BRI and their impacts may be the key drivers of eco-environmental risks [90,91,92]. Infrastructure project implementation, such as the construction of roads, railway, pipelines and seaports, has affected several terrestrial and marine biodiversity hotspots, wilderness areas and other key protected areas, contributing to biodiversity loss and vegetation loss, due to habitat degradation, fragmentation and illegal activities such as poaching and logging [29,31,93,94]. Meanwhile, the BRI may drive water and soil pollution, as well as climate change, due to the Chinese investments in some energy exploitation projects and construction and maintenance of transportation infrastructures [89,90,95]. It may also accelerate the overexploitation of natural resources, such as water, energy and mineral resources in BRI participating countries, bringing eco-environmental challenges [96].

Faced with the potential negative impacts on the eco-environment, China has emphasized the importance of implementing the concept of green development into the implementation of the BRI [97]. Given that most BRI participating countries are developing countries with relevant low-income levels, they are confronted with sharp contradictions between socioeconomic development and eco-environmental protection. Therefore, it is necessary for China and BRI-related countries to strengthen cooperation and take the path of green development under the framework of BRI [97]. In response, in 2017, China came up with an improved version of the BRI, the “Greening BRI”, aiming to support the BRI’s green development, achieve the Paris Agreement and promote the Sustainable Development Goals (SDGs) [89,98]. The Green BRI aims to promote green infrastructure, green investment and green finance, which have become the common needs of BRI-related countries and China to realize green development [99,100].

To reduce environmental deterioration and improve environmental quality, a great number of infrastructure projects with high environmental protection standards under the Green BRI have been introduced, decreasing the discharging of pollutants and making the eco-environment less prone to degradation [101]. Based on previous studies, the Green BRI could improve the environmental quality of BRI-participating countries by boosting their technological progress, tightening their environmental regulation, increasing the proportion of green energy consumption, promoting clean energy, upgrading industrial structures as well as investing in new energy projects rather than traditional oil and gas projects [89,99,102]. Green BRI is proven to be a feasible plan to support green and low-carbon development, guarantee biodiversity as well as address climate change, thus protecting the eco-environment [90]. With the growing global agenda on green development and the recognition of global initiatives and plans (such as the SDGs and the Paris Agreement) and climate change action plans, it is likely that the emerging role of the BRI with green development connotations will be more imperative in protecting the eco-environment and promoting global green and sustainable development. Therefore, it is worthwhile to explore feasible schemes to optimize the contribution of the BRI to the eco-environment from more aspects, improve the eco-environment in Belt and Road regions as well as enhance the capacity and effectiveness of the BRI.

## 5. Prospects for Future Studies Related to Ecological Environment in the BRI Regions

With the in-depth implementation of BRI, especially in the case of severe global climate change, certain impacts and challenges brought by external factors will have negative effects on the eco-environment and natural resources in the BRI regions. However, systematic, holistic and coordinated research on eco-environmental issues in BRI regions remains insufficient. Fundamental scientific research on eco-environmental issues in BRI regions with wider coverage and interdisciplinary approaches should be prioritized to lay a foundation for the expansion of in-depth research. Additionally, in response to the impacts and challenges on the eco-environment, the Green BRI, aiming at avoiding the conflict between economic development and eco-environmental protection, has been proven to be a viable plan to support green and low-carbon development, guarantee biodiversity, and address climate change. Consequently, it is worthwhile to carry out more integrated, interdisciplinary and coordinated research on eco-environmental issues in BRI regions, so as to explore feasible schemes to optimize the contribution of the BRI on the eco-environment from more aspects and improve the eco-environment in Belt and Road regions. On this basis, the following research directions could be considered in future research.

### 5.1. Comparative Study before and after the Implementation of the BRI

Due to the unstable ecological base and the vigorous development of the social economy, the current ecological environment of the BRI region is fragile. Tracking the dynamics and changes in natural resources and the eco-environment since the implementation of the BRI in 2013 and comparing the situation before and after the implementation are of great significance. In this way, the environmental problems needing to be addressed in each region and the driving factors behind the changes could be examined and identified, providing a reference to adjusting and optimizing the implementation plan of the BRI to maximize its contribution to the environment. Meanwhile, a holistic study could be conducted to find out the significant differences between regions under the same implementation plan, with the distinctness and varieties being targeted for further detailed analysis.

### 5.2. Research on Ecological Environmental Laws and Policies

#### 5.2.1. Research on Ecological Environmental Laws of BRI-Related Countries

Ecological environmental law is essential to coordinating the relationship between socioeconomic development and environmental protection, maintaining ecological balance and harmonizing the development of human society and nature as well. Studying and revealing the characteristics of eco-environmental laws in BRI member countries will contribute to forming a more suitable and scientific sustainable development design for realizing the complementarity of the BRI and local laws, facilitating the efficient development of the local social economy without destroying the eco-environment.

#### 5.2.2. Ecological Environmental Policies Evaluation

Aiming to judge the benefit, efficiency, effect and value of the policy, policy evaluation is an important link in the process of policy operation, which is directly related to the revision, adjustment and re-selection of policies. The policies of many BRI member countries on eco-environmental governance have fallen behind international standards, bringing unsatisfactory results. Therefore, in the future, the effect of the implementation of eco-environmental policies would be evaluated according to the distinctive conditions of BRI participating countries, before decisions are made on the continuation, revision or termination of policies. Additionally, the relationship between the implementation of the eco-environmental policies of the target country and that of relevant policies of the BRI could be examined to figure out potential mutual influence, which is conducive to adjusting the implementation plan of the BRI to improve eco-environment and achieve fruitful results. It is also a worthwhile direction to judge how local ecological environmental policies can complement the BRI, so as to ensure the efficient implementation of policies and contribute to the stability of the eco-environment with the rapidly developing economy.

### 5.3. Research on Natural Resources Complementary among BRI Participating Countries

BRI-related countries are rich in natural resources, including water resources, mineral resources, fossil energy (such as coal, crude oil and natural gas) and forest resources, but they are confronted with uneven distribution characteristics and insufficient utilization. It is important to explore more comprehensive bilateral and multilateral relations among BRI participating countries and tap the potential of resource complementarity and cooperation among them, which would give full play to the resource advantages of all member countries, guarantee the proper and efficient resource flows and enable in-depth cooperation among BRI participating countries for sustainable development.

### 5.4. Research on the Expansion Area

The BRI is a sustainable development initiative; therefore, it is necessary to conduct research related to the eco-environment of the Belt and Road region from the perspective of development. The expansion of the cooperation area should also be taken into account. By strengthening the research in the expanded area, a more systematic and comprehensive understanding of the eco-environment of the BRI member countries could be achieved, and a more comprehensive, appropriate and scientific design for the sustainable development of the BRI could be put forward. For instance, evaluating where additional natural resources are likely to be found in the expansion area could be carried out to relieve pressure on resource exploitation in early participating countries. In addition, mutually engaging in eco-environment protection in the expansion zone can also attract other countries in the region to participate in the BRI framework, contributing to achieving broader global cooperation.

### 5.5. Study on the Impact of International Emergencies

International emergencies, such as the COVID-19 epidemic, the conflict between Russia and Ukraine and other great power competitions, might affect international cooperation and the smooth implementation of the BRI. Therefore, the impact of international emergencies is to be considered in future research on the Belt and Road eco-environment to ensure ecological stability along the routes.

### 5.6. Interdisciplinary Research

Compared with a single discipline with clear boundaries in the past, interdisciplinary disciplines can provide more diverse theoretical foundations and perspectives, which are more likely to produce creative results. Interdisciplinary study, such as the combination of the research methods of natural science with those of humanities and social sciences, is needed to reveal the overall ecological environment of the Belt and Road region and promote the BRI’s sustainable development. Future research could focus on the underlying impact of regional culture, history and politics on the eco-environment of the Belt and Road region, such as examining the interaction effect of the control of the corruption and income levels on environmental quality in BRI regions and addressing these issues with natural science measures under the framework of the BRI. Meanwhile, with each region having its own characteristics at different levels of economic development, the eco-environmental research of the BRI regions should also take distinctive socio-economic factors and geological features into consideration.

## 6. Conclusions

Having summarized the eco-environment conditions in different BRI-related regions through a literature review, we found that most regions along the BRI routes are in sensitive zones of climate and geological change, with fragile eco-environments, and strong vulnerability to climate change, natural disasters and human activities. Therefore, it is understandable that the quest for socioeconomic development under the BRI brings some inevitable challenges to the eco-environment.

In the early stage of implementation of the BRI, infrastructure development, trade and investments under the BRI might put some stress on the regional environment. However, the situation has improved since the BRI was upgraded. It is worthwhile to explore feasible schemes to optimize the contribution of the green BRI on the eco-environment from more aspects and maximize its potential. On this basis, the possible future research directions are proposed for more systematic and holistic research: (1) Tracking the dynamics and changes of natural resources and the eco-environment since the implementation of the BRI to identify the environmental problems needing to be addressed in each region and reckon its drivers. (2) Studying and revealing the features of eco-environmental laws and policies in BRI participating countries to adjust the implementation plan of the BRI to achieve fruitful results. (3) Achieving the natural resources complementary among BRI-related countries to guarantee proper and efficient resource flows and in-depth cooperation among participating countries. (4) Extending the research scope to the expanded area with a more holistic and scientific design for the sustainable development of the BRI. (5) Weighing the impact of international emergencies on the BRI cooperation and its influence on the regional eco-environment. (6) Integrating different disciplines to explore the underlying factors affecting the eco-environment in the BRI region.

With adequate scientific research, careful planning, sufficient data and close cooperation amongst the participating countries, the BRI can be developed in a sound and environmentally friendly way, contributing to tackling environmental challenges and fulfilling the Sustainable Development Goals (SDGs) throughout the BRI countries by 2030.

## Figures and Tables

**Table 1:** Timetable and specific routes for the Belt and Road Initiative.

Proposal	Time (Location)	Specific Routes
Silk Road Economic Belt	September 7, 2013 (in Kazakhstan, Central Asia)	(1) Connecting China to Europe and the Baltic Sea (via Central Asia and Russia). (2) Connecting China to the Persian Gulf, and the Mediterranean (through Central Asia and West Asia). (3) Connecting China to the Indian Ocean (via the Indo-China Peninsula).
21st Century Maritime Silk Road	October 3, 2013 (in Indonesia, Southeast Asia)	(1) From Chinese coastal ports to Europe (via the South China Sea and the Malacca Strait to the Indian Ocean). (2) From Chinese coastal ports to the South Pacific (via the South China Sea).

Source: summarized by authors according to official documents.

**Table 2:** List of BRI-involved countries.

Region	Time	Country
Asia	2014	Qatar, Sri Lanka, Kazakhstan
	2015	Korea, Turkey, Iraq, Azerbaijan, Georgia, Armenia, Tajikistan, Uzbekistan
	2016	Cambodia, Laos, Bangladesh, Iran, Afghanistan
	2017	Mongolia, Singapore, Timor-Leste, Malaysia, Myanmar, Vietnam, Brunei, Pakistan, Nepal, Maldives, Lebanon, Thailand
	2018	United Arab Emirates, Kuwait, Oman, Saudi Arabia, Bahrain, Kyrgyzstan, Indonesia, Philippines
	2019	Yemen
	2022	Syria
Africa	2016	Egypt
	2017	Morocco, Madagascar
	2018	Sudan, South Africa, Senegal, Sierra Leone, Côte d’Ivoire, Somalia, Cameroon, South Sudan, Seychelles, Guinea, Ghana, Zambia, Mozambique, Gabon, Namibia, Mauritania, Angola, Djibouti, Ethiopia, Kenya, Nigeria, Chad, Congo, Zimbabwe, Algeria, Tanzania, Burundi, Cabo Verde, Uganda, Gambia, Togo, Rwanda, Tunisia, Libya
	2019	Equatorial Guinea, Liberia, Lesotho, Comorin, Benin, Mali, Niger
	2021	The Democratic Republic of the Congo, Botswana, Central Africa, Guinea-Bissau, Eritrea, Burkina Faso, Sao Tome and Principe
	2022	Malawi
Europe	2014	Belarus
	2015	Russia, Poland, Serbia, Czech Republic, Bulgaria, Slovakia, Hungary, North Macedonia
	2017	Albania, Croatia, Bosnia and Herzegovina, Montenegro, Estonia, Lithuania, Slovenia, Romania, Latvia, Ukraine, Moldova
	2018	Austria, Greece, Malta, Portugal
	2019	Italy, Luxembourg, Cyprus
Oceania	2017	New Zealand
	2018	Papua New Guinea, Samoa, Niue, Fiji, The Federated States of Micronesia, Cook Islands, Tonga, Vanuatu
	2019	Solomon Islands
	2020	Kiribati
South America	2018	Chile, Guyana, Bolivia, Uruguay, Venezuela, Surinam, Ecuador
	2019	Peru
	2022	Argentina
North America and the Caribbean	2017	Panama
	2018	Costa Rica, El Salvador, Dominica, Trinidad and Tobago, Antigua and Barbuda, Dominica, Grenada
	2019	Barbados, Cuba, Jamaica
	2022	Nicaragua

Source: Belt and Road Portal (https://www.yidaiyilu.gov.cn/xwzx/roll/77298.htm, accessed on 20 July 2022).

**Table 3:** Classification of the BRI-involved countries up to 2022.

Category	Income Group	Country
The Silk Road Economic Belt region	High income (17)	Austria, Cyprus, Czech Republic, Croatia, Estonia, Greece, Poland, Slovakia, Slovenia, Lithuania, Hungary, Romania, Latvia, Malta, Portugal, Italy, Luxembourg
	Upper middle income (15)	Turkey, Iraq, Armenia, Azerbaijan, Albania, Bulgaria, Bosnia and Herzegovina, Belarus, Georgia, Kazakhstan, Russia, Serbia, Montenegro, North Macedonia, Moldova
	Lower middle income (6)	Mongolia, Iran, Kyrgyzstan, Tajikistan, Uzbekistan, Ukraine
	Low income (2)	Afghanistan, Syria
The 21st Century Maritime Silk Road region	High income (10)	Korea, Singapore, Brunei, United Arab Emirates, Kuwait, Qatar, Oman, Saudi Arabia, Bahrain, Seychelles
	Upper middle income (9)	Malaysia, Maldives, Thailand, South Africa, Gabon, Namibia, Libya, Equatorial Guinea, Botswana
	Lower middle income (33)	Timor-Leste, Myanmar, Cambodia, Vietnam, Laos, Pakistan, Sri Lanka, Bangladesh, Nepal, Lebanon, Indonesia, Philippines, Senegal, Côte d’Ivoire, Cameroon, Ghana, Mauritania, Angola, Djibouti, Kenya, Nigeria, Congo, Zimbabwe, Algeria, Tanzania, Cabo Verde, Morocco, Tunisia, Egypt, Lesotho, Comoros, Benin, Sao Tome and Principe
	Low income (25)	Yemen, Sudan, Sierra Leone, Somalia, South Sudan, Guinea, Zambia, Mozambique, Ethiopia, Chad, The Democratic Republic of the Congo, Burundi, Uganda, Gambia, Togo, Rwanda, Madagascar, Liberia, Mali, Niger, Central Africa, Guinea-Bissau, Eritrea, Burkina Faso, Malawi
The natural extension region of the Maritime Silk Road	High income (7)	New Zealand, Chile, Uruguay, Panama, Trinidad and Tobago, Antigua and Barbuda, Barbados
	Upper middle income (16)	Fiji, Niue, Cook Islands, Tonga, Guyana, Venezuela, Surinam, Ecuador, Peru, Argentina, Costa Rica, Dominica, Dominican, Grenada, Cuba, Jamaica
	Lower middle income (9)	Papua New Guinea, Samoa, The Federated States of Micronesia, Vanuatu, Solomon Islands, Kiribati, Bolivia, El Salvador, Nicaragua

Source: summarized by authors according to the World Bank categorization (https://data.worldbank.org/country, assessed on 22 July 2022).

**Table 4:** The spatial precipitation levels distribution in the Belt and Road region.

Precipitation Level	Belt and Road Region
The highest level (Annual precipitation: 1867 mm)	South-Eastern Asia
The moderate level	Mongolia-Russia; Central Asia; Central and Eastern Europe
The lowest level (Annual precipitation: 142 mm)	Western Asia; Northern Africa

Source: data from Ref. [35].

**Table 5:** Regional characteristics of natural disasters in the Silk Road Economic Belt region.

Region	Natural Disaster Type
Southeast Asia	Floods; landslides; winter drought
South Asia	Floods; landslides; drought; earthquakes
West Asia	Floods; landslides; winter drought; earthquakes
Central Asia	Floods; earthquakes; landslides; summer drought
Central and Eastern Europe	Floods; landslides
The Middle East	Earthquakes; floods; droughts
North Africa	Floods; landslides; summer drought

Source: adapted from the results of Refs. [35,39,41,45,46].

**Table 6:** The mean annual NDVI of some regions.

The Mean Annual NDVI	Region
Above 0.7	South Asia
0.4–0.7	East Europe; Russia; South China
Below 0.2	Central Asia; West Asia

Source: data from Ref. [51].

**Table 7:** Water resources change in the Belt and Road region and countries under climate change.

Changing Situation	Region and Countries
Decreasing water resources	Central and West Asia, South Africa, South China, Chile, Bolivia and Venezuela
Increasing water resources	North Africa, Russia, Mongolia Plateau, West China, Southeast Asia, Peru and Uruguay

Source: adapted from the results of Ref. [34].

**Table 8:** Types and contents of mineral resources in the Belt and Road region.

Resources Type	Content (Proportion)
The total recoverable oil	203.64 Gt (Accounting for 66% of the global total)
The total recoverable natural gas	204.2 × 1012 m^3^ (Accounting for 65.5% of the global total)
The total mining areas	24,234 km^2^ (Accounting for 42.31% of the global mining areas)

Source: data from Refs. [69,75,76].

## Data Availability

Not applicable.
